# Population Aging and Multicultural Diversity in the United States: Implications for Older Consumers’ Needs and Expectations

**DOI:** 10.1093/geroni/igab046.915

**Published:** 2021-12-17

**Authors:** Julie Miller, Taylor Patskanick, Lisa D'Ambrosio, Joseph Coughlin

**Affiliations:** 1 MIT, Cambridge, Massachusetts, United States; 2 MIT, Somerville, Massachusetts, United States; 3 Massachusetts Institute of Technology, Cambridge, Massachusetts, United States

## Abstract

Looking ahead to 2030 and beyond, the United States will be both older and more multicultural than presently. To explore the impacts and characteristics of an increasingly diverse population beginning to age, the MIT AgeLab conducted online focus groups in August 2020 (n=92) with ethnically diverse participants ages 40-69 on topics related to household composition, use of technology and digital engagement. Regarding household composition, Black and Latinx participants were more likely to report living with grandchild(ren), and Asian, Latinx, and White participants were more likely to report living with a parent(s) or parent(s)-in- law. Latinx participants often described ways in which caregiving for aging parents was a cultural value, but many participants who had raised children in the United States but who were not born in the United States themselves described cultural gaps in family attitudes that had sometimes widened across the generations. While all participants were using some technology, due to the coronavirus pandemic, digital tools were being used more widely than ever before. Racial/ethnic identity groups were more similar than different in terms of their responses to questions around consumer digital engagement. There were notable differences in overall trust in technology across racial/ethnic groups, with Asian participants reporting the highest average overall level of trust in technology and Multiracial participants reporting the lowest. Looking ahead, the intersection of aging and growing racial/ethnic diversity in the United States will yield a wider array of consumer needs and expectations.

